# Diagnostic accuracy of the Mini-Balance Evaluation Systems Test (Mini-BESTest) in screening for fall risk among individuals with vestibular disorders

**DOI:** 10.1371/journal.pone.0353230

**Published:** 2026-07-08

**Authors:** Nichaporn Khumduang, Kanokporn Pooranawatthanakul, Nattawan Utoomprurkporn, Akkradate Siriphorn

**Affiliations:** 1 Department of Physical Therapy, Faculty of Allied Health Sciences, Chulalongkorn University, Bangkok, Thailand; 2 Otoneurology Unit, Otolaryngology Department, Faculty of Medicine, Chulalongkorn University, Bangkok, Thailand; Children Hospital Lahore: University of Child Health Sciences, PAKISTAN

## Abstract

Vestibular disorders significantly impair postural stability and elevate fall risk, necessitating the use of validated assessment tools. While the Mini-Balance Evaluation Systems Test (Mini-BESTest) is a comprehensive measure of balance, diagnostic cut-off scores tailored to the vestibular population remain poorly defined. This study aimed to evaluate the diagnostic accuracy of the Mini-BESTest in identifying fall risk among individuals with vestibular disorders. Forty-two participants underwent assessment using the Mini-BESTest, 4-Meter Walk Test (4MWT), and Timed Up and Go (TUG) test. Fall risk was classified using a dual-criteria approach: (1) fall history and severity (e.g., injury, frequency, and frailty) and (2) physical performance benchmarks on the 4MWT and TUG. Participants were subsequently categorized into low or intermediate-to-high fall risk groups. Accuracy was determined via receiver operating characteristic (ROC) curve analysis. The results yielded an Area Under the Curve (AUC) of 0.69, indicating poor-to-modest discriminatory ability. At the optimal cut-off score of ≤ 23, the scale demonstrated high sensitivity (92.3%) and low specificity (44.8%), resulting in a positive likelihood ratio (LR+) of 1.67 and a negative likelihood ratio (LR-) of 0.17. Exploratory subscore analysis revealed that only the reactive postural control domain provided significant discriminatory trends under bootstrap analysis (95% CI: 0.516–0.823). These results suggest that while the comprehensive total score of Mini-BESTest remains the necessary standard for primary risk stratification due to the scale’s validated unidimensional design, reactive postural control performance represents a key underlying physiological driver of instability in this population. Recognizing this specific domain impairment can better assist clinicians in tailoring targeted, individualized therapeutic interventions during vestibular rehabilitation.

## Introduction

The vestibular system, located in the inner ear, is essential for regulating balance and perceiving spatial orientation. Dysfunction within this complex network can lead to vestibular disorders, which significantly impair balance and mobility [1]. Epidemiological studies report varying prevalence rates, including 6.5% in Germany [2], 2.98% in the U.S. [3], and a significant 2.83% in Taiwan [4], with the elderly population experiencing rates as high as 18.6% [5].

Vestibular disorders are broadly categorized into peripheral and central types. Peripheral vestibular disorders result from damage to the vestibular apparatus itself, while central vestibular disorders originate within the central nervous system and are often accompanied by additional neurological symptoms [1]. The clinical manifestations are diverse and may include vertigo, dizziness, nausea, hearing loss, tinnitus, aural fullness, oscillopsia, photophobia, phonophobia, and visual aura [6–8]. These symptoms disrupt postural control and heighten fall risk, with serious consequences for quality of life and safety [9–11].

Accurate fall risk assessment is essential in this population. Common clinical tools used to evaluate balance include the Berg Balance Scale, Dynamic Gait Index, and Timed Up and Go (TUG) Test. However, these assessments often lack sensitivity to critical components of postural control, such as sensory integration and reactive balance responses. For example, the TUG test evaluates functional mobility but does not capture adaptive or sensory strategies [12]. In contrast, the original Balance Evaluation Systems Test (BESTest) was developed to assess all systems contributing to balance control. Despite its comprehensiveness, the BESTest’s length and complexity limit its routine clinical use [13].

To address these limitations, the Mini-Balance Evaluation Systems Test (Mini-BESTest) was created as a shortened version of the BESTest. It includes 14 items that examine four domains of balance: anticipatory postural adjustments, reactive postural control, sensory orientation, and dynamic balance during gait. This format allows for efficient yet comprehensive assessment of both static and dynamic stability and advanced sensorimotor functions [14]. The Mini-BESTest has demonstrated good psychometric properties and has been used in older adults [15], individuals with Parkinson’s disease [16], and post-stroke populations [17].

While validated Mini-BESTest cut-off scores for fall risk have been established in other neurological and geriatric populations, no study to date has established a definitive diagnostic cut-off score optimized specifically for predicting fall risk within individuals with vestibular disorders. Previous vestibular studies have restricted their use of the Mini-BESTest to characterizing balance impairments or tracking longitudinal rehabilitation outcomes [18,19]. For instance, Zhu et al. (2023) mapped exploratory ROC curves comparing patients with bilateral vestibulopathy to healthy controls, reporting raw sensitivity and specificity coordinates across the entire score spectrum without designating or recommending a specific clinical cut-off score [18].

Accordingly, this study aimed to assess the diagnostic accuracy of the Mini-BESTest in distinguishing between low and intermediate-to-high fall risk among individuals with vestibular disorders. While the Mini-BESTest is widely recognized as a unidimensional scale for assessing dynamic balance [20], an exploratory analysis of its subsections, i.e., anticipatory postural adjustments, reactive postural control, sensory orientation, and dynamic gait, was also conducted to identify the specific postural control domains most predictive of fall risk in this population. Such insights could ultimately enhance clinical decision-making through more targeted rehabilitation assessments.

## Materials and methods

### Study design

This cross-sectional study enrolled 42 individuals diagnosed with vestibular disorders from King Chulalongkorn Memorial Hospital, Bangkok, Thailand. Participants were recruited using consecutive sampling from eligible individuals attending the clinic between 18/11/2025 and 30/01/2026. The study protocol was approved by the Research Ethics Review Committee for Research Involving Human Research Participants, Group I, Chulalongkorn University (Approval No. 320/68). All participants provided written informed consent prior to participation. This study is reported in accordance with the Standards for Reporting Diagnostic Accuracy (STARD) guidelines (S2 File) to ensure transparent and complete reporting of the study’s design, conduct, and results.

### Participants

Participants were eligible for inclusion if they met the following criteria: aged between 40 and 80 years; experiencing chronic dizziness and/or unsteadiness for more than three months; and diagnosed with either peripheral or mixed (peripheral and central) vestibular disorders by a neuro-otologist, following the Barany Society diagnostic criteria. Additional inclusion criteria were the ability to ambulate independently for at least 10 meters, normal visual acuity (with or without corrective lenses), and a Montreal Cognitive Assessment (MoCA) score of 23 or higher [21].

Exclusion criteria included: the presence of lower limb injury or pain (rated ≥2 on a numerical pain rating scale) that could affect gait or balance, and severe dizziness or intolerance during balance testing procedures.

### Sample size

The required sample size was calculated using MedCalc® version 23.2.6. Parameters for the calculation included a Type I error (α) of 0.05, a Type II error (β) of 0.10, an expected area under the curve (AUC) of 0.80, a null hypothesis value of 0.50, and a 2:1 ratio of participants classified as negative (low fall risk) to positive (intermediate-to-high fall risk). Based on these assumptions, the minimum required sample size was 39 participants (26 in the negative group and 13 in the positive group). To maintain adequate statistical power and account for an anticipated 7% dropout rate, a total of 42 participants were enrolled.

### Procedure

Eligible participants were first screened using a structured questionnaire and the MoCA, with a minimum score of 23 required to ensure adequate cognitive function. After providing written informed consent, participants completed a standardized interview and underwent a series of physical assessments. Demographic and clinical information—including age, sex, body mass index (BMI), medical history, symptom duration, vestibular symptoms, and fall history over the previous year—was collected. To ensure consistency during testing, participants were asked to remove their shoes and socks, and all procedures were conducted using standardized instructions.

Fall risk classification was determined through a two-step approach. The first step involved assessment of fall history and fall severity, including whether the participant had experienced any falls in the past year, sustained fall-related injuries, had difficulty rising unassisted after a fall, experienced any loss of consciousness, or reported more than two falls in the previous 12 months. Frailty status was also evaluated using the Fried Frailty Phenotype. In the second step, functional mobility and gait performance were assessed using the 4-Meter Walk Test (4MWT) and the TUG Test. Based on the combined results of these two steps, participants were categorized into either a low fall risk group or an intermediate-to-high fall risk group, following international fall risk screening guidelines [22]. This dual-criteria approach was chosen because retrospective fall history can be prone to recall bias and may fail to identify ‘pre-fallers’—individuals who maintain a negative fall history only by strictly limiting their mobility [22]. By incorporating objective physical performance benchmarks (TUG and 4MWT), we established a more robust clinical reference standard for functional instability in this population. The index test (Mini-BESTest) was performed on the same day as the reference standard assessments, with no clinical interventions occurring between the two. To minimize potential bias, two licensed physical therapists independently conducted the assessments. One assessor collected demographic and clinical data, and administered the fall severity, 4MWT, and TUG assessments. The second assessor, blinded to the participant’s classification and prior results, independently conducted the Mini-BESTest.

### Assessments

#### Fall history and fall severity.

Fall history was assessed based on participant self-report of any falls occurring within the past year. Fall severity was evaluated using multiple criteria, including whether the participant sustained an injury during the fall, was able to rise unassisted from the ground, experienced a loss of consciousness, or had more than two falls in the past 12 months. In addition, frailty was assessed using the Fried Frailty Phenotype [23]. Participants were classified as frail if they met at least three of the following five criteria: (1) unintentional weight loss of more than 4.5 kg in the past year; (2) self-reported exhaustion, based on two items from the CES-D Depression Scale (“I felt that everything I did was an effort” and “I could not get going”), with a response of 2 or higher (moderate or frequent) to either item considered positive; (3) low physical activity, assessed using the Thai version of the short-form International Physical Activity Questionnaire (IPAQ) [24]; (4) slow walking speed was determined using a 4.5-meter walk test, with cut-off values adjusted for gender and height. For male participants, a time of ≥7 seconds indicated slow gait if height was ≤ 173 cm, and >6 seconds if height was > 173 cm. For female participants, a time of ≥7 seconds was used for those with height ≤159 cm, and >6 seconds for those taller than 159 cm.; and (5) reduced grip strength, stratified by gender and body mass index (BMI) quartiles, according to Fried Frailty Phenotype [23].

#### 4-Meter walk test (4MWT).

The 4MWT was used to assess steady-state gait speed, an established predictor of functional decline and fall risk. Participants were instructed to walk a total of 8 meters at their usual pace. Timing was recorded over the central 4 meters to ensure measurement during steady walking, excluding acceleration and deceleration phases. Gait speed was calculated in meters per second (m/s) using the measured time to complete the 4-meter segment. A cut-off value of <0.8 m/s was used to indicate high fall risk, in line with international guidelines for fall risk assessment [22].

#### Timed up and go test (TUG).

The Timed Up and Go (TUG) Test was used to evaluate functional mobility and dynamic balance. Participants were instructed to sit in a standard chair with armrests, rise upon a verbal cue, walk a distance of three meters at a comfortable pace, turn around, walk back, and return to a seated position. Timing began at the moment the participant’s back left the chair and stopped once they were fully seated again. A cut-off time of ≥11.1 seconds was used to indicate increased fall risk in individuals with vestibular disorders. This threshold was selected based on prior research specific to this population [25], rather than the more general cut-off values used for older adults.

#### Mini-balance evaluation systems test (Mini-BESTest).

The Mini-BESTest was used to assess multiple dimensions of postural control relevant to fall risk. It consists of 14 items grouped into four domains: anticipatory postural adjustments, reactive postural control, sensory orientation, and dynamic gait. Each item is scored on a 3-point scale (0–2), where 0 indicates severe impairment and 2 indicates normal performance. The total score ranges from 0 to 28, with higher scores reflecting better balance.

The anticipatory postural adjustment domain includes tasks such as sit-to-stand, rising onto the toes, and standing on one leg. The reactive postural control domain evaluates compensatory stepping responses to external perturbations in forward, backward, and lateral directions. Sensory orientation is assessed through standing in different sensory conditions, including firm and foam surfaces and inclined surfaces with eyes open or closed. The dynamic gait domain includes tasks such as walking with speed changes, head turns, pivot turns, obstacle negotiation, and a dual-task version of the TUG. The Mini-BESTest was administered using standardized instructions and typically took 10–15 minutes to complete [14].

### Statistical analysis

Statistical analyses were performed using MedCalc® version 23.2.6. Descriptive statistics summarized participant characteristics. The Shapiro-Wilk test assessed data distribution. The MoCA score in the intermediate-to-high fall risk group, along with height, symptom duration, physical activity, 4MWT, and Mini-BESTest scores in both the low and intermediate-to-high fall risk groups, were not normally distributed. Consequently, group comparisons were conducted using the independent samples t-test for variables with a normal distribution and the Mann-Whitney U test for non-normally distributed variables. Chi-square tests compared nominal variables, including gender, vestibular disorder type, history of falls, and frailty status. Statistical significance was set at p < 0.05. Receiver operating characteristic (ROC) curve analysis evaluated the ability of parameters to distinguish between low and intermediate-to-high fall risk. The Area Under the Curve (AUC) was interpreted according to the following criteria: ≥ 0.90 indicated excellent accuracy, 0.80–0.89 indicated considerable accuracy, 0.70–0.79 indicated fair accuracy, 0.60–0.69 indicated poor accuracy, and < 0.60 indicated a failure to discriminate [26]. The Mini-BESTest and its subscores were analyzed as continuous variables to assess their discriminatory performance using ROC analysis. No pre-specified cut-off scores were applied; cut-offs were considered exploratory and derived from data-driven analysis where applicable. The optimal Mini-BESTest cut-off was identified using Youden’s index (J).

To further assess clinical utility, the sensitivity, specificity, positive predictive value (PPV), negative predictive value (NPV), and diagnostic odds ratio (DOR) were calculated for the total score and each subscore. Positive and negative likelihood ratios were calculated to further assess diagnostic accuracy, with interpretation based on established thresholds. Likelihood ratios were interpreted as follows: an LR+ greater than 10 or an LR- less than 0.1 was considered highly informative for clinical decision-making (very useful); values of LR+ between 5 and 10 or LR- between 0.1 and 0.2 were moderate utility; LR+ between 2 and 5 or LR- between 0.2 and 0.5 provided limited utility; and LR+ between 1 and 2 or LR- between 0.5 and 1 were not considered clinically useful (minimal utility) [27]. No indeterminate or missing results occurred for either the index test (Mini-BESTest) or the reference standard assessments. All participants completed the full protocol.

To ensure internal validation and robustness of the estimates, 1,000 bootstrap resampling iterations were performed. Bootstrap methods were used to calculate confidence intervals for the AUC, the Youden index, and the optimal cut-off scores. A *p*-value of less than 0.05 was considered statistically significant. No analyses of variability in diagnostic accuracy across subgroups were performed. All diagnostic accuracy assessments were exploratory and applied to the overall sample.

## Results

**Fig 1:**
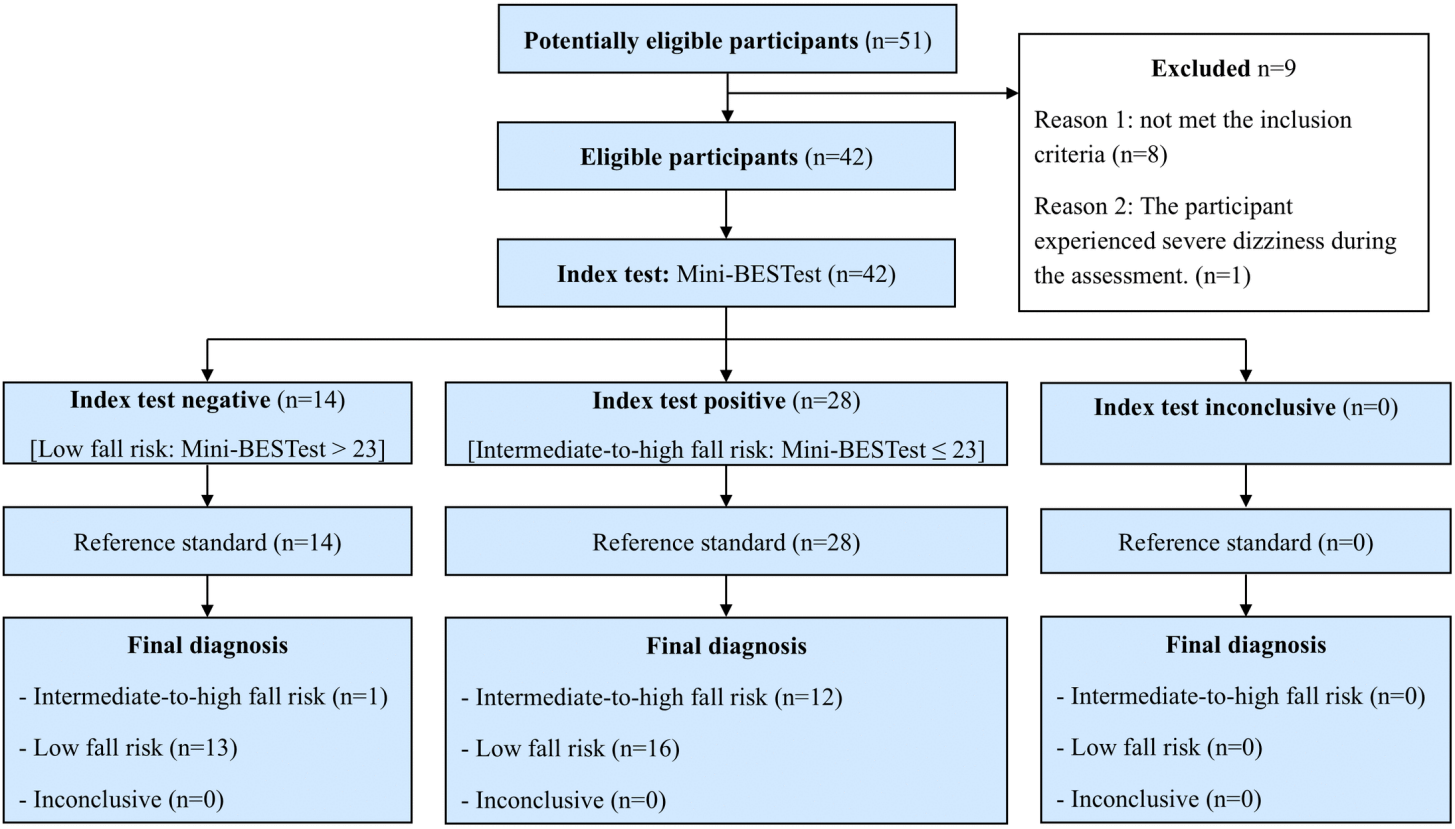
Flow of participants in the study.

Fifty-one potential participants were screened for this study, of whom forty-two met the inclusion criteria and were enrolled. Nine participants were excluded for not meeting the eligibility requirements. One participant experienced dizziness during testing and was excluded from further assessment for safety reasons. No other adverse events occurred during administration of the Mini-BESTest or reference standard assessments. The flow of participants is illustrated in Fig 1. The characteristics of the participants and the results of all outcome measures are summarized below.

### Characteristic data of the participants

Participant characteristics stratified by fall risk group are summarized in Table 1. No significant differences were found between the low and intermediate-to-high fall risk groups in terms of age, sex, BMI, vestibular diagnosis, symptom duration, MoCA score, physical activity level, or physical performance tests (4MWT and TUG).

**Table 1:** Participant characteristics stratified by low fall risk and intermediate-to-high fall risk groups.

Characteristics	Low fall risk (n = 29)	Intermediate-to-high fall risk (n = 13)	*p*-value
Gender, n (%) Female Male	23 (79.3%) 6 (20.7%)	8 (61.5%) 5 (38.5%)	0.270 ^c^
Age (years)	65.62 ± 8.76	66.00 ± 6.61	0.260 ^a^
Weight (kg)	56.78 ± 11.77	62.31 ± 16.85	0.085 ^a^
Height (cm)	158.53 ± 7.95	157.46 ± 10.67	0.376 ^b^
Body mass index (kg/m²)	22.58 ± 4.21	24.72 ± 3.82	0.304 ^a^
Vestibular disorders, n (%)			
Peripheral vestibular disorders	14 (48.3%)	8 (61.5%)	0.559 ^c^
Episodic syndromes	6 (20.7%)	3 (23.1%)	
Functional / Others	9 (31.0%)	2 (15.4%)	
Duration of symptoms (years)	5.07 ± 7.16	5.77 ± 8.24	0.978 ^b^
Montreal Cognitive Assessment (MoCA) score	25.41 ± 1.68	24.54 ± 1.27	0.093 ^b^
History of fall, n (%)			
Faller	4 (13.8%)	13 (100%)	< 0.001 ^c,^*
Non-faller	25 (86.2%)	0 (0%)	
Frailty, n (%)			
Robust	12 (41.4%)	2 (15.4%)	0.138 ^c^
Pre-frail	16 (55.2%)	9 (69.2%)	
Frail	1 (3.4%)	2 (15.4%)	
Thai-IPAQ (kcal/week)	1,741.88 ± 2,875.73*	836.31 ± 1,031.67	0.453 ^b^
4-meter walk test (4MWT) (m/s)	1.15 ± 0.44	1.44 ± 1.56	0.479 ^b^
Timed up and go test (TUG) (s)	11.36 ± 2.14	11.36 ± 1.47	0.297 ^a^
Mini-BESTest			
Total score	22.41 ± 2.71	20.08 ± 4.01	0.049 ^b,^*
• Anticipatory postural adjustment	5.00 ± 0.80	4.46 ± 1.13	0.116 ^b^
• Reactive postural control	4.10 ± 1.59	3.46 ± 1.33	0.042 ^b,^*
• Sensory orientation	5.34 ± 0.67	5.08 ± 0.64	0.207 ^b^
• Dynamic gait	7.97 ± 1.21	5.08 ± 0.64 7.08 ± 1.80	0.093 ^b^

NOTE: Values are mean ± SD; n (%). Vestibular categories are defined as: 1) Peripheral (BPPV, Vestibular Neuritis, Meniere’s Disease, Vestibular Schwannoma, and Bilateral Vestibulopathy); 2) Episodic (Vestibular Migraine and Meniere’s Disease); 3) Functional/Other (PPPD and unspecified disorders). BPPV, Benign Paroxysmal Positional Vertigo; PPPD, Persistent Postural-Perceptual Dizziness; Thai-IPAQ, Thai Version of Short Format International Physical Activity Questionnaire; ^a^ Independent samples *t*-test; ^b^ Mann-Whitney U test, ^c^ Chi-square tests; *Statistical significance at *p* < 0.05.

However, fall history differed significantly between the groups, with all individuals in the intermediate-to-high risk group reporting at least one fall in the past year (*p* < 0.001). Additionally, significant differences were observed in the Mini-BESTest total score (*p* = 0.049) and the reactive postural control subscore (*p* = 0.042), suggesting potential value in differentiating fall risk levels using these balance measures (Table 1).

### Receiver Operating Characteristic (ROC) curve

**Fig 2:**
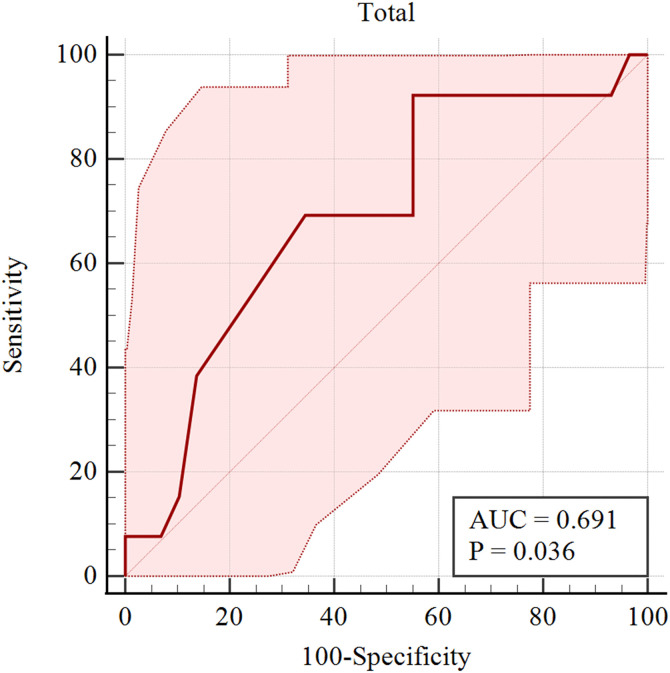
Receiver operating characteristic (ROC) curve analysis evaluating the diagnostic accuracy of the Mini-BESTest total score in identifying fall risk. The red shaded area represents the 95% confidence interval for the area under the curve (AUC = 0.69, *p* = 0.036). The diagonal reference line indicates the line of no discrimination (AUC = 0.50).

The Mini-BESTest demonstrated a significant ability to discriminate between low and intermediate-to-high fall risk groups among individuals with vestibular disorders, with an AUC of 0.69 (95% CI: 0.530–0.824) (Fig 2 and Table 2). The optimal cut-off score was ≤ 23, yielding a high sensitivity of 92.31% (95% CI: 64.0–99.8) but a relatively low specificity of 44.83% (95% CI: 26.4–64.3). The positive predictive value (PPV) was 42.9%, and the negative predictive value (NPV) was 92.9%. The diagnostic odds ratio (DOR) for the total score was 9.82. The positive likelihood ratio (LR+) was 1.67 (95% CI: 1.16–2.41), and the negative likelihood ratio (LR–) was 0.17 (95% CI: 0.03–1.18), as shown in Table 2.

**Table 2:** AUC, cut-off score, sensitivity, specificity, LR + , and LR– of total score and subscores (anticipatory postural adjustments, reactive postural control, sensory orientation, and dynamic gait) for predicting fall risk in persons with vestibular disorders classified into low fall risk and intermediate-to-high fall risk groups.

	Total score	Subscores			
		Anticipatory postural adjustment	Reactive postural control	Sensory orientation	Dynamic gait
AUC	0.69	0.65	0.69	0.61	0.66
Standard error	0.09	0.10	0.08	0.08	0.10
95% CI	0.530-0.824	0.482-0.786	0.528-0.823	0.449-0.758	0.497-0.798
95% Bootstrap CI^a^	0.491-0.836	0.488-0.838	0.516-0.823	0.455-0.738	0.463-0.818
P-value	0.036*	0.131	0.018*	0.187	0.103
Youden index J	0.371	0.305	0.363	0.218	0.382
95% Bootstrap CI^a^	0.154-0.520	0.072-0.562	0.122-0.578	0.037-0.457	0.127-0.639
Cut-off score	≤23	≤4	≤4	≤5	≤7
95% Bootstrap CI^a^	≤21 to ≤25	≤2 to ≤5	≤3 to ≤5	≤4 to ≤5	≤5 to ≤9
Sensitivity (95% CI)	92.31 (64.0-99.8)	61.54 (31.6-86.1)	84.62 (54.6-98.1)	76.92 (46.2-95.0)	69.23 (38.6-90.9)
Specificity (95% CI)	44.83 (26.4-64.3)	68.97 (49.2-84.7)	51.72 (32.5-70.6)	44.83 (26.4-64.3)	68.97 (49.2-84.7)
LR+ (95% CI)	1.67 (1.16-2.41)	1.98 (0.99-3.96)	1.75 (1.13-2.73)	1.49 (0.28-7.86)	2.23 (1.16-4.28)
LR- (95% CI)	0.17 (0.03-1.18)	0.56 (0.27-1.16)	0.30 (0.08-1.12)	0.94 (0.73-1.23)	0.45 (0.19-1.05)
PPV (%)	42.9%	47.1%	44.0%	38.5%	50.0%
NPV (%)	92.8%	80.0%	88.2%	81.3%	83.3%
DOR	9.82	3.54	5.83	1.58	4.95

Note: AUC: area under the ROC curve; SE: standard error; LR + : positive likelihood ratio; LR-: negative likelihood ratio; PPV: positive predictive value; NPV: negative predictive value; DOR: Diagnostic Odds Ratio.

*Statistical significance at p < 0.05.

a Bias-corrected and accelerated (BCa) bootstrap confidence interval (1,000 iterations; random number seed: 978).

**Fig 3:**
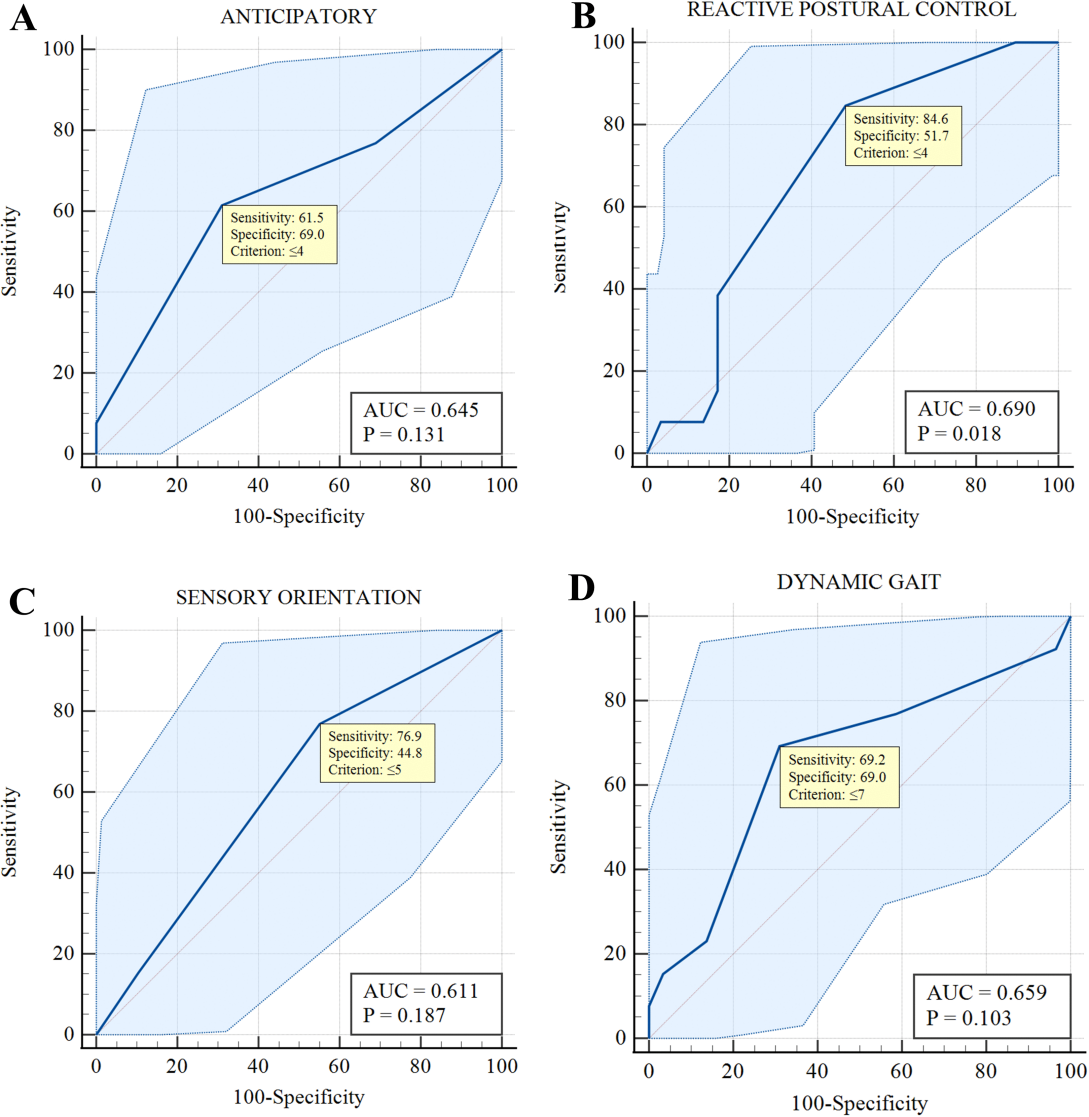
Receiver operating characteristic (ROC) curve analysis for the four domains of the Mini-BESTest: (A) Anticipatory Postural Adjustments, (B) Reactive Postural Control, (C) Sensory Orientation, and (D) Dynamic Gait. The Reactive Postural Control subscore (B) was the only domain to demonstrate statistically significant discriminatory power (AUC = 0.690, *p* = 0.018) with an optimal cut-off of ≤ 4. Blue shaded areas represent the 95% confidence intervals for each area under the curve.

Among the four Mini-BESTest subscores, anticipatory postural adjustments (*p* = 0.131), sensory orientation (*p* = 0.187), and dynamic gait (*p* = 0.103) did not demonstrate statistically significant discrimination between low and intermediate-to-high fall risk groups (Fig 3 and Table 2). In contrast, the reactive postural control subscore showed moderate diagnostic accuracy, with an AUC of 0.69 (95% CI: 0.528–0.823; *p* < 0.05), indicating its utility in fall risk classification for individuals with vestibular disorders.

## Discussion

This study aimed to evaluate the accuracy of the Mini-BESTest in identifying fall risk among individuals with vestibular disorders. The findings indicated a significant difference between groups with low and intermediate-to-high fall risk as determined by the Mini-BESTest. Of the subscores, only reactive postural control demonstrated significant discriminatory ability, whereas anticipatory postural adjustments, sensory orientation, and dynamic gait did not. Notably, while both the total score and the reactive postural control subscore exhibited similar accuracy, only the reactive postural control subscore remained statistically significant when assessed using the bootstrap method. These results suggest that while the comprehensive total score remains the standard for clinical risk stratification due to the scale’s fundamental unidimensionality, reactive postural control performance may be a key underlying physiological driver of balance failure in this population. This underscores its potential clinical utility for guiding targeted therapeutic interventions rather than functioning as a standalone diagnostic threshold.

The general characteristics of the low fall risk and intermediate-to-high fall risk groups were largely comparable, with the exception of fall history. As anticipated, a significantly greater proportion of individuals in the intermediate-to-high fall risk group reported a history of previous falls, which is consistent with existing evidence that fall history is a strong predictor of future risk [28]. Additionally, in this study, fall risk groups were classified based in part on fall history. As a result, participants with no history of falls were automatically assigned to the low fall risk group. Therefore, it is not surprising that there was a clear difference in fall history between the low and intermediate-to-high fall risk groups. Differences in characteristics such as MoCA scores and symptom duration were likely indicative of the heterogeneous nature of the vestibular disorder population. Additionally, differences in time since diagnosis may have affected balance performance, as the sample included both newly diagnosed individuals and those with chronic symptoms. This diversity is representative of recruitment from routine outpatient care, where patients are at varying stages of diagnosis and recovery.

Individuals with vestibular disorders exhibited reduced balance performance compared to healthy individuals [1,9–11,18]. This can be attributed to the vestibular system, which is one of the key systems involved in regulating postural stability [29–32]. The vestibular system is essential for sensing head movements and spatial orientation, as well as integrating sensory information necessary for maintaining balance [1]. When this system becomes dysfunctional, the brain receives distorted or inadequate input regarding body position and movement, thereby hindering the ability to execute appropriate postural adjustments [1,31]. Consequently, these individuals experience impaired postural control and face a higher risk of imbalance and falls [31,33].

The Mini-BESTest scores differed significantly between the low and intermediate-to-high fall risk groups. Specifically, the total Mini-BESTest score demonstrated poor discriminatory ability in distinguishing between these groups among individuals with vestibular disorders, with an area under the curve (AUC) of 0.69. Although this finding was statistically significant, the level of accuracy was lower than initially anticipated. The optimal cut-off score was identified as ≤ 23, resulting in high sensitivity (92.31%) but low specificity (44.83%). This high sensitivity suggests the tool may be useful for ruling out fall risk; however, the low specificity indicates a high rate of false positives in this population. To our knowledge, this was the first study to evaluate the accuracy of the Mini-BESTest for detecting fall risk specifically in individuals with vestibular disorders. Previous research in other populations has shown variable accuracy. For example, among individuals with chronic stroke, the Mini-BESTest demonstrated similarly low accuracy (AUC = 0.64; cut-off = 17.5; sensitivity = 64.0%; specificity = 64.2%) [17]. Additionally, moderate accuracy was observed in older adults (AUC = 0.71; cut-off = 18; sensitivity = 91%, specificity = 62%) [15], individuals with Parkinson’s disease (AUC = 0.75; cut-off = 19; sensitivity = 79%, specificity = 67%) [16], and nursing home residents (AUC = 0.71; cut-off = 9.0; sensitivity = 77.8%, specificity = 70.9%) [34]. These findings suggested that, although the Mini-BESTest could be useful for initial fall risk screening in individuals with vestibular disorders, its modest accuracy and limited specificity indicate that it should not be used as a standalone definitive assessment. Instead, it should be supplemented with additional clinical information to achieve more reliable risk stratification.

It is important to note that structural psychometric evaluations of the Mini-BESTest strongly support its unidimensional nature, proving that the cumulative total score reflects a single underlying construct of dynamic balance control [20]. While individual subsections target clinically distinct physical mechanisms, evaluating independent cut-offs for them can be psychometrically problematic due to this overarching unidimensionality. Nevertheless, our exploratory analysis suggests that specific domains may drive their underlying clinical utility in certain tracking environments. Specifically, the reactive postural control subscore of the Mini-BESTest demonstrated substantial utility as a descriptive measure of an individual’s capacity to promptly reestablish equilibrium following unexpected external disturbances through rapid, compensatory actions [32]. Optimal recovery of stability in response to unexpected perturbations depends on the seamless integration of visual, somatosensory, and vestibular sensory inputs within the reactive postural control system [32]. This function was frequently compromised in individuals with vestibular disorders as a result of disrupted sensory input from the vestibular system, which impaired the body’s ability to perceive and appropriately respond to alterations in spatial orientation [1,35]. Consequently, these individuals exhibited a reduced capacity to perform timely and effective balance corrections, which contributed to an elevated risk of falling [36,37]. For instance, fallers typically required a greater number of recovery steps following perturbations compared to non-fallers [36]. These findings underscored the clinical significance of the reactive postural control subscore in fall risk assessment for this population. In contrast, the other subscores, anticipatory postural adjustments, sensory orientation, and dynamic gait, did not effectively discriminate between fall risk categories in current study.

The Mini-BESTest total score demonstrated high sensitivity (92.31%), which is highly desirable for primary screening purposes, as it enables the identification of most individuals at risk of falling. Conversely, the low specificity (44.83%) indicates that the tool is less effective for confirming or diagnosing true cases of fall risk in isolation. Clinically, this suggests that while the Mini-BESTest can be valuable tool for initial fall risk screening in individuals with vestibular disorders, it should be complemented by additional assessments to improve diagnostic accuracy and guide targeted interventions.

Beyond sensitivity and specificity, the clinical utility of the Mini-BESTest is best illustrated by its Predictive Values and the DOR. In our cohort, the total score (≤ 23) yielded a high NPV of 92.9%. This indicates that the Mini-BESTest is an excellent ‘rule-out’ tool; a patient scoring above 23 has less than an 8% probability of being at high risk for falls. Conversely, the modest PPV of 42.9%—driven by the tool’s lower specificity—suggests that a positive screen (≤23) should be interpreted as a ‘red flag’ requiring further clinical validation rather than a definitive diagnosis of fall risk.

Furthermore, the DOR of 9.82 for the total score demonstrates a strong overall diagnostic power, as the odds of testing positive are nearly ten times higher in true ‘high-risk’ individuals than in ‘low-risk’ individuals.

The present study found that the LR+ for the Mini-BESTest total score was 1.67, while the LR- was 0.17. Generally, an LR+ greater than 1 indicates that a positive test result is more likely among individuals at high risk of falling than those at low risk; however, values less than 2 are typically regarded as having limited diagnostic value [27]. Likewise, LR− values below 1 suggest that a negative test result is associated with a lower probability of high fall risk, but values above 0.1–0.2 offer only minimal confidence in ruling out risk [27]. Accordingly, the Mini-BESTest total score exhibited only modest discriminatory power in differentiating between individuals at low and intermediate-to-high fall risk. These findings suggest that, while the Mini-BESTest can contribute to fall risk assessment, its overall clinical utility as a standalone screening tool in individuals with vestibular disorders remains limited.

This was the first study to examine the accuracy of the Mini-BESTest in identifying fall risk among individuals with vestibular disorders. Few previous investigations have assessed the diagnostic accuracy of balance assessments for predicting fall risk in this population. Prior research indicated that the TUG test, using a cut-off score of 11.1 seconds, achieved moderate accuracy with a sensitivity of 80.0%, specificity of 55.6%, and an area under the curve (AUC) of 0.71 [25]. Additionally, the Dynamic Gait Index demonstrated low accuracy, with a cut-off score of 18, a sensitivity of 64.5%, a specificity of 63.4%, and an AUC of 0.67 [25]. Furthermore, while Zhu et al. (2023) mapped exploratory ROC curves for the Mini-BESTest in a vestibular cohort, their work was restricted to diagnostic discrimination against healthy controls rather than defining a prognostic fall-risk cut-off [18]. Although the Mini-BESTest in this study demonstrated slightly lower overall accuracy (AUC = 0.69) compared to the TUG, it provided a broader scope of assessment. While tools like the TUG or 4MWT primarily capture basic functional mobility or gait speed, the Mini-BESTest systematically challenges distinct motor control systems, including anticipatory adjustments, sensory orientation, and dynamic gait adaptability. Notably, our exploratory bootstrap analysis revealed that the reactive postural control subscore was the sole domain to maintain statistical significance, underscoring its unique physiological relevance in vestibular instability. Despite a modest overall threshold accuracy, the comprehensive nature of the complete Mini-BESTest enables clinicians to identify specific system impairments to better tailor targeted, individualized interventions in clinical settings.

A major strength of this study was that it was the first to evaluate the accuracy of the Mini-BESTest in detecting fall risk among individuals with vestibular disorders, thereby providing a clinically relevant cut-off score for balance assessment in this population. Furthermore, this study investigated the individual subscores of the Mini-BESTest providing comprehensive clinical profile data regarding distinct balance domains. Rather than suggesting that subscores be used as independent diagnostic metrics in isolation—which would conflict with the scale’s validated unidimensional structure—identifying that reactive postural control performance acts as a primary physiological driver of variance allows clinicians to better understand the nature of vestibular instability. This dual focus on a total screening threshold alongside exploratory subscore characterization provides a valuable framework for both initial risk screening and subsequent targeted rehabilitation planning.

Despite these promising findings, several limitations should be noted. The overrepresentation of female participants may have limited the generalizability of the results to male populations; however, this gender imbalance was consistent with established epidemiological patterns observed in vestibular disorders [38,39]. Furthermore, this study did not incorporate symptom-specific measures. Previous research demonstrated that individuals who reported higher levels of dizziness-related distress tended to walk more slowly and had lower confidence in their balance [40], both of which could have affected their performance on the Mini-BESTest and influenced its predictive accuracy. Methodologically, our two-step reference standard included the TUG and 4MWT, which share movement components with the Mini-BESTest. This potential incorporation bias may lead to an overestimation of the reported AUC. However, it is important to distinguish the underlying constructs: while the TUG and 4MWT measure functional mobility and walking speed, the Mini-BESTest challenges higher-level vestibular integration through tasks like head turns and pivot turns. These items specifically evaluate the adaptability of gait under sensory challenge, whereas the reference standard assesses basic performance. Therefore, while the mode of activity (walking) overlaps, the sensorimotor demands differ significantly between the index test and the reference standard. Additionally, because this was a cross-sectional study, the ‘fall risk’ classification serves as a surrogate for actual falling events. While this model successfully captures functional decline, reliance on self-reported fall history may have introduced recall bias. Finally, while the sample size (n = 42) is relatively small, we employed Bootstrap resampling (1,000 iterations) as a robust method for internal validation. This approach ensures the stability and reliability of our diagnostic estimates (AUC, sensitivity, and specificity) and serves as a statistically rigorous alternative to split-sample cross-validation, which is often unfeasible in smaller clinical cohorts. This approach mitigates the risks associated with small sample distributions. Nevertheless, the resulting AUC of 0.69 suggests that the Mini-BESTest possesses poor-to-modest discriminatory ability in this population, and future large-scale studies are encouraged to further validate these cut-off scores.

Future studies should focus on validating these findings in more diverse populations and examining whether integrating symptom-specific and patient-reported instruments, such as the Dizziness Handicap Inventory, enhances the accuracy of fall risk assessment. To address the limitations of the current reference standard, implementing prospective tracking of actual falls over a 6-to-12-month period is essential to establish true predictive validity. Lastly, future studies comparing the Mini-BESTest with other balance assessments, while incorporating both objective and subjective measures, may provide a more comprehensive framework for evaluating fall risk in individuals with vestibular disorders.

For clinical implications, these findings indicate that clinicians should exercise caution when relying exclusively on the Mini-BESTest to assess fall risk in this population, due to its limited accuracy. Furthermore, given limitations such as the gender imbalance, absence of symptom-specific measures, and use of fall history data obtained from participant self-report, it is advisable to adopt a comprehensive and multifaceted strategy for evaluating fall risk. Such an approach should encompass objective balance assessments, patient-reported outcome measures, and consideration of additional risk factors, including frailty and psychological well-being. Considering the demonstrated importance of reactive postural control in fall risk, future investigations should examine whether interventions specifically targeting this domain can effectively decrease the incidence of falls among individuals with vestibular disorders.

## Conclusions

This study found that while the Mini-BESTest could significantly distinguish between low and intermediate-to-high fall risk groups among individuals with vestibular disorders, its overall diagnostic accuracy was poor-to-modest. Subscores for anticipatory postural adjustments, sensory orientation, and dynamic gait did not show significant discriminatory ability. In contrast, the reactive postural control subscore was the sole domain to maintain statistical significance under bootstrap analysis, highlighting its distinct physiological relevance to instability in this cohort. Therefore, while the comprehensive total score remains the necessary standard for primary clinical risk stratification due to the scale’s validated unidimensional design, clinicians should pay close attention to reactive postural control performance to guide targeted therapeutic interventions. Despite their limited specificity, the Mini-BESTest remains a valuable clinical tool for initial screening due to its high sensitivity in detecting individuals at risk of falling.

## Supporting information

S1 Table Raw Data. This file contains the anonymized individual participant data, including Mini-BESTest total and subscores, used for the ROC analysis and the calculation of sensitivity, specificity, and predictive values.
(XLSX)

S1 File STARD Checklist. Completed STARD 2015 checklist for reporting diagnostic accuracy studies.
(DOCX)
